# Evaluation of Biodegradation of BTEX in the Subsurface of a Petrochemical Site near the Yangtze River, China

**DOI:** 10.3390/ijerph192416449

**Published:** 2022-12-08

**Authors:** Xuexia Chen, Shuai Zhang, Lijin Yi, Zhengwei Liu, Xiangyu Ye, Bo Yu, Shuai Shi, Xiaoxia Lu

**Affiliations:** 1 Ministry of Education Laboratory for Earth Surface Processes, College of Urban and Environmental Sciences, Peking University, Beijing 100871, China; 2 State Key Laboratory of Safety and Control for Chemicals, SINOPEC Research Institute of Safety Engineering Co., Ltd., Qingdao 266100, China

**Keywords:** BTEX, biodegradation, soil, groundwater, microcosm, electron acceptor

## Abstract

The contamination of soil and groundwater with BTEX (benzene, toluene, ethyl benzene, and xylenes) is a common issue at petrochemical sites, posing a threat to the ecosystems and human health. The goal of this study was to evaluate the biodegradation of BTEX in the subsurface of a petrochemical site near the Yangtze River, thus providing scientific basis for bioremediation of the contaminated site. Both molecular analysis of field samples and microcosm study in the laboratory were performed for the evaluation. Soil and groundwater samples were collected from the site. Microcosms were constructed with inoculum from the soil and incubated anaerobically in the presence of nitrate, ferric oxide, manganese oxide, sulfate, and sodium bicarbonate, respectively. The initial concentration of each component of BTEX (benzene, toluene, ethyl benzene, o-xylene) was 4–5 mg/L. Actinobacteria was dominant in the highly contaminated soil, while Proteobacteria was dominant in the slightly contaminated soil and the groundwater. The relative abundances of Firmicutes, Spirochaetes, and Caldiserica were higher in the highly contaminated soil and groundwater samples compared to those in the corresponding slightly contaminated samples. The relative abundances of predicted functions, such as carbohydrate transport and metabolism, nucleotide transport and metabolism, coenzyme transport and metabolism, amino acid transport and metabolism, etc., in the highly contaminated soil and groundwater samples were higher than those in the corresponding slightly contaminated samples. In microcosms, biodegradations of BTEX occurred, and the first-order rate constants in the presence of various electron acceptors had the following order: sulfate (0.08–0.10/d) > sodium bicarbonate (0.07–0.09/d) > ferric oxide (0.04–0.06/d) > nitrate (0.03–0.05/d) > manganese oxide (0.01–0.04/d).

## 1. Introduction

The Yangtze River Economic Belt carries 30% of the petrochemical industry in China, and most of these enterprises, such as oil refineries, oil depots, and gas stations, are distributed along the Yangtze River. During their activities, soil and groundwater contamination may occur due to leakage of production facilities, storage tanks, underground pipelines, etc., posing threats to the ecosystem and human health of the Yangtze River basin [1,2].

The pollutants of petrochemical enterprises consist of hundreds of compounds. Among them, BTEX (benzene, toluene, ethylbenzene, o-xylene, m-xylene, and p-xylene) and PAHs (polycyclic aromatic hydrocarbons) are the most concerning ones due to their high toxicities [3]. Compared to PAHs, BTEX are more easily transported to deep soil and groundwater due to their weak hydrophobicity [4]. Zhai et al. investigated a petrochemical enterprise in the northeast area of China and found that soil was contaminated with BTEX and PAHs and that the carcinogenic risk of benzene, ethylbenzene, and naphthalene in the soil was beyond the 1 × 10^−6^ levels acceptable to human health [5]. Sun et al. carried out an investigation of groundwater pollution in the surrounding area of a coastal petrochemical enterprise in North China, and the results showed that petroleum hydrocarbons, including benzene, were detected in some wells [6].

In order to mitigate the risks associated with BTEX, their removal from the soil and groundwater has become an important issue. Several techniques, such as soil vapor extraction, air sparging, chemical oxidation, and bioremediation, have been developed for removing BTEX from contaminated sites [7]. In comparison with other techniques, bioremediation has received more attention due to its cost-effective, energy-efficient, and environmentally sound attributes [8].

In soil and groundwater with long-term contamination, the oxygen level is usually very low or zero due to consumption of oxygen by microorganisms [9]. Therefore, understanding anaerobic biodegradation of BTEX is critical for bioremediation of contaminated soil and groundwater. Extensive research efforts have been devoted to the anaerobic biodegradation of BTEX, and BTEX-degrading bacteria, pathways, and genes have been characterized [10,11,12,13,14,15,16,17,18,19,20,21]. The results show that many factors may influence the biodegradation of BTEX, including concentrations of BTEX, environmental conditions, and microorganisms. This study aimed to characterize the microbial communities of soil and groundwater collected from a petrochemical site near the Yangtze River and to compare the biodegradation of BTEX in the presence of various electron acceptors. The results may provide scientific basis for remediation and risk control of the contaminated site.

## 2. Materials and Methods

### 2.1. Site Description and Sampling

**Figure 1:**
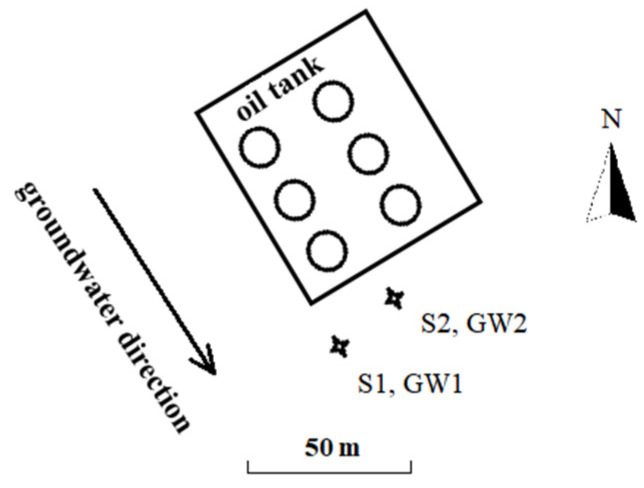
Locations of the soil and groundwater sampling points (S indicates soil, and GW indicates groundwater).

The studied petrochemical site is located on the bank of the middle lower reach of the Yangtze River, China. Contaminations of soil and groundwater were found in an area close to the oil tanks, which mainly contain gasoline. The groundwater table was 3–5 m below the ground. In September (post-rain season), two soil samples (S1 and S2) were collected at about 4.0 m below the ground during the construction of the monitoring wells using Geoprobe (Geoprobe Systems^®^, Salina, CA, USA). Groundwater samples (GW1 and GW2) in the monitoring wells were collected using Baylor Tube after the construction of the wells. Baylor tube is a grab sampler. Sampling processes included the following steps: sampler being lowered to depth to fill with groundwater, sampler being raised to surface, checking if the ball retained the groundwater as the sampler was lifted, and loading the groundwater into containers. The locations of the sampling points are shown in Figure 1.

### 2.2. Analyses of Physiochemical Properties of the Collected Samples

For the soil sample, the pH, conductivity, total nitrogen, and total phosphate were measured using a soil testing instrument (Puruisenshe, Jinan, China). To measure pH, the pH electrode was inserted into the soil and equilibrated for 1 min. The number in the meter was then read. The concentrations of C6–C9, C10–C40, and volatile organic compounds (VOC) were measured following the standard methods issued by the Ministry of Ecology and Environmental Protection of China [22,23,24].

For the groundwater sample, the pH, conductivity, ORP, DO, chemical oxidation demand (COD), nitrite, sulfide, iron, and manganese were measured using a water quality analyzer (S500A, Beijing, China). The concentrations of VOC were measured following the standard methods issued by the Ministry of Ecology and Environmental Protection of China [25].

### 2.3. Analyses of Microbial Communities of the Collected Samples

For the soil sample, 0.5 g soil was used for DNA extraction. For groundwater sample, 500 mL groundwater was filtered onto a 0.22 µm membrane, and the membrane was used for DNA extraction. PowerSoil^®^ DNA kit (MO BIO Laboratories, San Diego, CA, USA) was used to extract DNA, the integrity of DNA was detected by 1% agarose gel electrophoresis, and the concentration and purity of DNA were determined by NanoDrop one (Thermo Scientific™, Waltham, MA, USA). Bacterial specific primers 338F (5′-ACTCCTACGGGAGGCAGCA-3′) and 806R (5′-GGACTACHVGGGTWTCTAAT-3′) with 12 bp barcode were used to amplify 16S rRNA gene in V3–V4 region [26,27]. PCR products were mixed in equidensity ratios according to the GeneTools analysis software (Version 4.03.05.0, SynGene). Then, the mixture PCR products were purified with E.Z.N.A Gel Extraction Kit (Omega Bio-tek, Norcross, GA, USA). The NEBNext^®^ Ultra™ DNA Library Prep Kit for Illumina^®^ standard procedures were followed for library construction. The constructed amplicon library was sequenced for PE250 using Illumina Hiseq2500 platform. After the paired-end raw reads data filtering, paired-end clean reads stitching, and raw tags sequence quality filtering, effective clean tags were obtained. Clean tags of all samples were clustered by usearch software (V8.0.1517). The sequences were clustered into OUT (operational taxonomic units) with 97% identity by default, and the default clustering method was uparse. The sequence with the highest frequency was used as the representative sequence of each OTU for subsequent annotation to obtain the community composition information of each sample. Phylogenetic Investigation of Communities by Reconstruction of Unobserved States 2 (PICRUSt 2) was used to predict the metagenome function for the microbial communities [28].

### 2.4. Microcosm Setup and Analyses

Benzene, toluene, ethylbenzene, and o-xylene (purity ≥ 99%) were purchased from Sigma, Germany. Other reagents (GR or AR) were purchased from Sinopharm, China. Basic culture medium was prepared according to the following composition: NH_4_Cl (1.0 g/L), MgCl_2_·7H_2_O (0.1 g/L), CaCl_2_·2H_2_O (0.05 g/L), yeast extract (0.1 g/L), KH_2_PO_4_ (0.90 g/L), Na_2_HPO_4_ (1.85 g/L), vitamin solution (10 mL/L), and mineral solution (0.001 mL/L). The components of vitamin solution and mineral solution are shown in Tables S1 and S2 in the Supplemental Materials. Resazurine was added (1 mg/L) as redox indicator. Soil microbial extract was prepared as follows: 25 g fresh soil was added to 1 L phosphate buffer (KH_2_PO_4_ 0.90 g/L, Na_2_HPO_4_ 1.85 g/L) purged with nitrogen, shaken well for 2 h on a shaker, and then left for 2 h. The supernatant was used as inoculum. Microcosms were set up using 100 mL serum bottles. In each microcosm, 60 mL of sterilized basic culture medium and 7 mL of soil microbial extract were added. The microcosms were divided into six treatments: five electron acceptor treatments with addition of sufficient nitrate, manganese oxide, ferric oxide, sulfate, and sodium bicarbonate and a blank control with no addition of electron acceptor but sterilized by autoclaving (under 121 °C for 30 min). All treatments were performed in triplicate. The medium was purged with nitrogen to remove oxygen, and the crimp was sealed. Next, 70 µL of BTEXstock solution was injected into the serum bottle using a glass syringe. All the microcosms were incubated in a temperature-controlled incubator (20 °C) at a shaking speed of 100 r/min. On day 0, 1, 7 and 12, water sample were collected from each microcosm to analyze BTEX.

The BTEX in samples were analyzed by headspace-based gas chromatograph (GC) carried out with an automated headspace sampler (Agilent 7694E) and a GC system (Agilent 7890A). Headspace operating conditions were as follows: low shaking of the tested sample solution at 75 °C for 1 min, GC cycle time = 27 min, vial pressurization time = 6 s, sample loop fill time = 30 s, and loop equal time = 3 s. The gas chromatograph was equipped with a capillary column (Agilent 19091J-413 0.32 mm × 30 m) and a flame ionization detector (FID). Injector, detector, and column temperatures were held at 150, 200, and 100 °C, respectively. Air and hydrogen served as fuel gas for the FID and nitrogen as carrier gas (flow rate = 4.0 mL/min).

The nominal concentration (total concentration of gas and liquid phases) was used to characterize the change of BTEX in the microcosms. The calculation formula was as follows:

M = (C_w_V_w_ + C_g_V_g_)/V_w_ = C_w_ (1 + H_c_ × V_g_/V_w_) (1)

where M is the nominal concentration (µg/L); C_w_ and C_g_ are the concentrations in water phase and gas phase (µg/L), respectively; V_w_ and V_g_ are the volumes of water phase and gas phase (mL), respectively; and H_c_ is Henry’s law constant (dimensionless).

### 2.5. Data Analyses

Qiime (V1.9.1) and R (V2.15.3) were used to analyze and map the microbial sequencing data. OriginPro (2021) was used to plot the BTEX data.

## 3. Results and Discussion

### 3.1. Characterization of the Soil and Groundwater Samples Collected from the Site

#### 3.1.1. Physicochemical Properties of the Samples

The physicochemical properties of the soil and groundwater samples collected from the studied petrochemical site are shown in Table 1 and Table 2. The results show that S1 was slightly contaminated, while S2 was highly contaminated. The pH of S2 was lower than that of S1, indicating that microbial degradation of the contaminants produced acid. In S2, the measured VOC consisted of 37.2% 1,2,4-trimethylbenzene, 15.3% ethylbenzene, 8.2% 1,3,5-trimethylbenzene, 6.6% m,p-xylene, 6.1% 1,1,2,2-tetrachloroethane, 4.2% isopropylbenzene, 3.8% benzene, 0.8% toluene, and 9.8% other compounds. The pH of the groundwater samples was neutral. The ORP and DO values indicated that the groundwater was in anaerobic condition. The detections of nitrite, sulfide, iron, and manganese in the groundwater indicated that different electron acceptor processes occurred in the site. The levels of contamination in the groundwater samples were consistent with those in the soil samples.

#### 3.1.2. Bacterial Community Structures of the Samples

**Figure 2:**
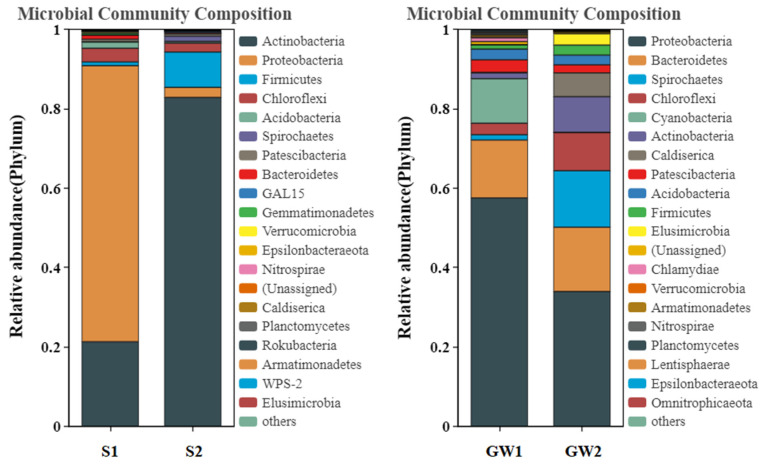
Comparisons of bacterial community structures at phylum level for the samples (S indicates soil, and GW indicates groundwater).

**Figure 3:**
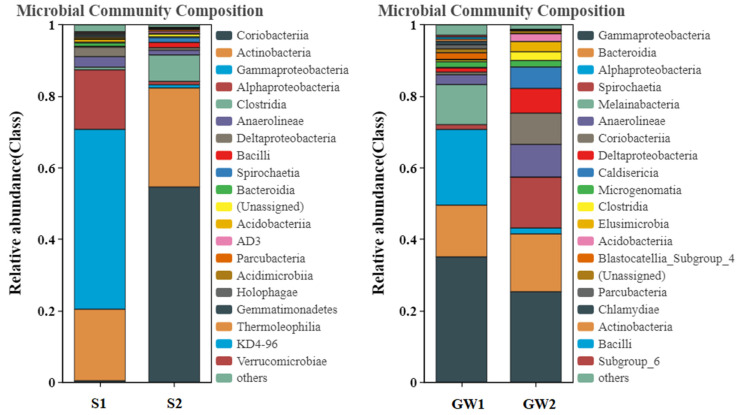
Comparisons of bacterial community structures at class level for the samples (S indicates soil, and GW indicates groundwater).

Via amplicon sequencing, the obtained numbers of sequences and OTUs as well as the calculated alpha diversity indices for the samples are shown in Table 3. For both soil and groundwater, the highly contaminated samples reduced the richness and diversity of bacterial community compared to the slightly contaminated samples. The bacterial community structures between the highly contaminated and slightly contaminated soil/groundwater samples were clearly quite different at various levels. Figure 2 and Figure 3 show the comparisons of bacterial community structures at the phylum and class levels.

At the phylum level, Proteobacteria was dominant (69.56%) in S1, while Actinobacteria was dominant (82.82%) in S2. The relative abundances of Firmicutes (8.92%), Spirochaetes (1.35%), and Caldiserica (0.06%) were higher in S2 compared to those in S1 (0.96%, 0.04%, and 0.02%). In both GW1 and GW2, Proteobacteria was dominant (57.49% and 33.91%), followed by Bacteroidetes (14.60% and 16.22%). Similar to the soil samples, the relative abundances of Spirochaetes (14.24%), Actinobacteria (8.98%), Caldiserica (5.99%), and Firmicutes (2.52%) were higher in GW2 compared to those in GW1 (1.36%, 1.45%, 0.12%, and 1.10%). However, the differences observed in the groundwater samples were less than those in the soil samples. In addition, unlike the soil samples, the relative abundances of Chloroflexi (9.61%) and Elusimicrobia (2.86%) in GW2 were higher than those in GW1 (2.88% and 0.01%).

At the class level, Gammaproteobacteria was dominant (50.30%) in S1, while Coriobacteriia was dominant (54.63%) in S2. The relative abundances of Actinobacteria (27.68%), Clostridia (7.40%), Spirochaetia (1.35%), Bacilli (1.47%), AD3 (0.54%), and Parcubacteria (0.50%) were higher in S2 compared to those in S1 (19.98%, 0,75%, 0.04%, 0.19%, 0.04% and 0,04%). In both GW1 and GW2, Gammaproteobacteria was dominant (35.08% and 25.33%), followed by Bacteroidia (14.46% and 16.17%). The relative abundance of Spirochaetia was much higher in GW2 (14.23%) compared to that in GW1 (1.36%). Unlike the soil samples, the relative abundances of Actinobacteria (0.16%), Bacilli (0.06%), and Parcubacteria (0.15%) were lower in GW2 compared to those in GW1 (0.63%, 0.59%, and 1.15%), while the relative abundances of Coriobacteriia (8.73%), Anaerolineae (9.13%), Caldisericia (5.99%), Deltaproteobacteria (6.92%), Elusimicrobia (2.86%), Acidobacteriia (2.20%), and Microgenomatia (1.82%) in GW2 were higher than those in GW1 (0.73%, 2.76%, 0.12%, 1.17%, 0.03%, 0.12%, and 1.60%). For both soil and groundwater, the relative abundances of Alphaproteobacteria in the highly contaminated samples (0.93% and 1.64%) were much lower than those in the slightly contaminated samples (16.64% and 21.17%), indicating Alphaproteobacteria was greatly inhibited by the contamination.

In previous studies, many strains of the Proteobacteria, Actinobacteria, Firmicutes and Chloroflexi phyla were found to be able to degrade aromatic hydrocarbons using nitrate, sulfate, and trivalent iron as electron acceptors [12,29]. Gammaproteobacteria and Deltaproteobacteria of Proteobacteria as well as Bacilli and Clostridia of Firmicutes played important roles in the anaerobic degradation of aromatic hydrocarbons [10,11,12,29,30,31]. BTEX biodegradation was found to be linked to bacterial community assembly patterns in contaminated groundwater ecosystem, where the relative abundances of Spirochaetes, Bacteroidetes, Firmicutes, Chloroflexi, and Deltaproteobacteria were higher compared to those in the deeper uncontaminated zone [32]. In this study, the relative abundances of the above-mentioned bacteria were also higher in the highly contaminated soil/groundwater samples as compared to the slightly contaminated samples. In addition, the relative abundance of Caldiserica was found to be higher in the highly contaminated samples, which has not been previously reported in the literature. This is a new discovery and further research is needed to explore the role of Caldiserica in the biodegradation of petroleum hydrocarbons.

#### 3.1.3. Functional Prediction for the Bacterial Communities

The COG family information corresponding to OTU was obtained by PICRUSt2. At the L2 level, the relative abundances of [G] carbohydrate transport and metabolism; [F] nucleotide transport and metabolism; [H] coenzyme transport and metabolism; [E] amino acid transport and metabolism; [J] translation, ribosomal structure, and biogenesis; [K] transcription; [L] replication, recombination, and repair; [D] cell cycle control, cell division, and chromosome partitioning; and [T] signal transduction mechanisms were all higher in sample S2/GW2 compared to those in sample S1/GW1, as shown in Figure S1.

**Figure 4:**
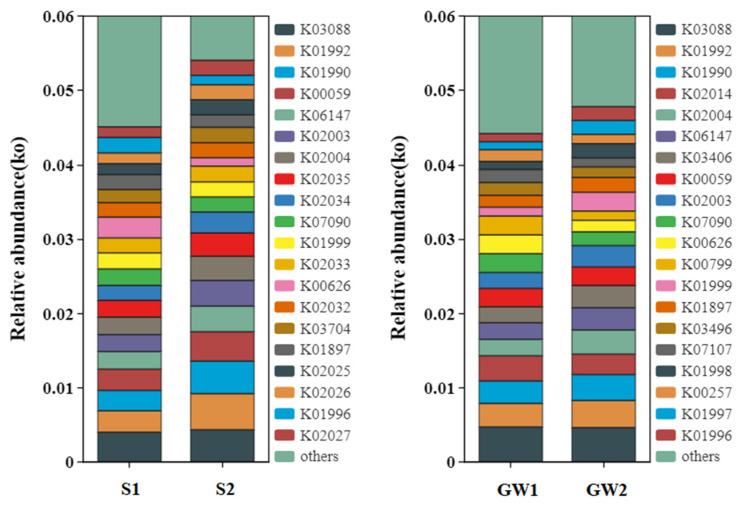
KEGG functional prediction at ko level for the samples (S indicates soil, and GW indicates groundwater).

Similar results were obtained by KEGG functional predictions using PICRUSt2. Figure 4 compares the top 20 ko between different samples. K02003, K02004, K06147, K01990, K01992, and K00059 in sample S2/GW2 had higher relative abundances than those in sample S1/GW1. K02003 is a putative ABC transport system ATP-binding protein, K02004 is a putative ABC transport system permease protein, K06147 is an ATP-binding cassette, K01990 is a ABC-2 type transport system ATP-binding protein, K01992 is a ABC-2 type transport system permease protein, and they are all involved in signaling and cellular processes. K00059 is a 3-oxoacyl- [acyl-carrier protein] reductase involved in many pathways, such as fatty acid biosynthesis, fatty acid biosynthesis, biotin metabolism, metabolic pathways, biosynthesis of secondary metabolites, fatty acid metabolism, and biosynthesis of cofactors. The relative abundance of K01897 was higher in sample GW2 compared to that in sample GW1. K01897 is an acyl-CoA synthetase involved in pathways such as fatty acid biosynthesis and quorum sensing. All the proteins were identified via the KEGG database. These proteins indicated that biodegradation of petroleum hydrocarbons were active in the site. For instance, propanoate metabolism and pyruvate metabolism were found to be the downgradient pathways of ethylbenzene degradation (Figure S2) [33]. Fatty acids must be converted into active fatty acyl CoA before oxidative decomposition, and acyl-CoA synthetase converted fatty acids to fatty acyl-CoAs by esterification [34].

### 3.2. Degradation of BTEX by the Soil Microorganisms

**Figure 5:**
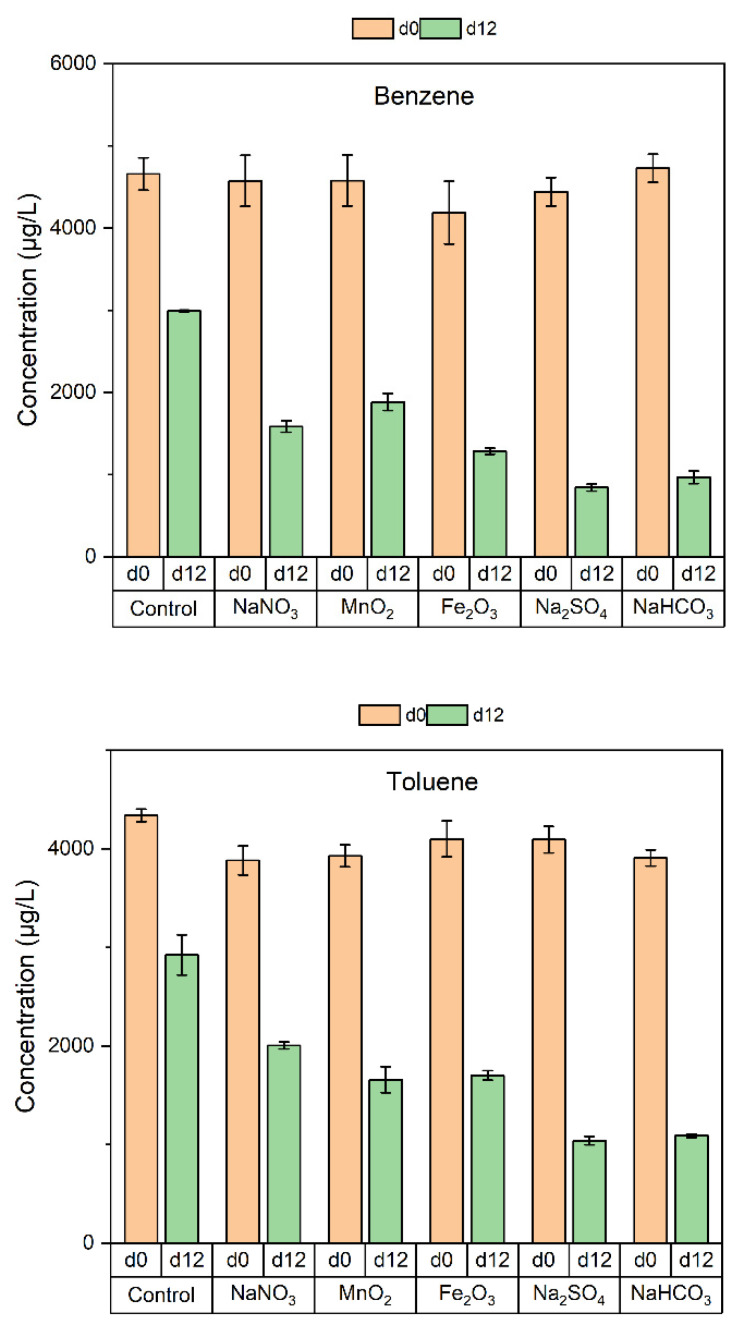

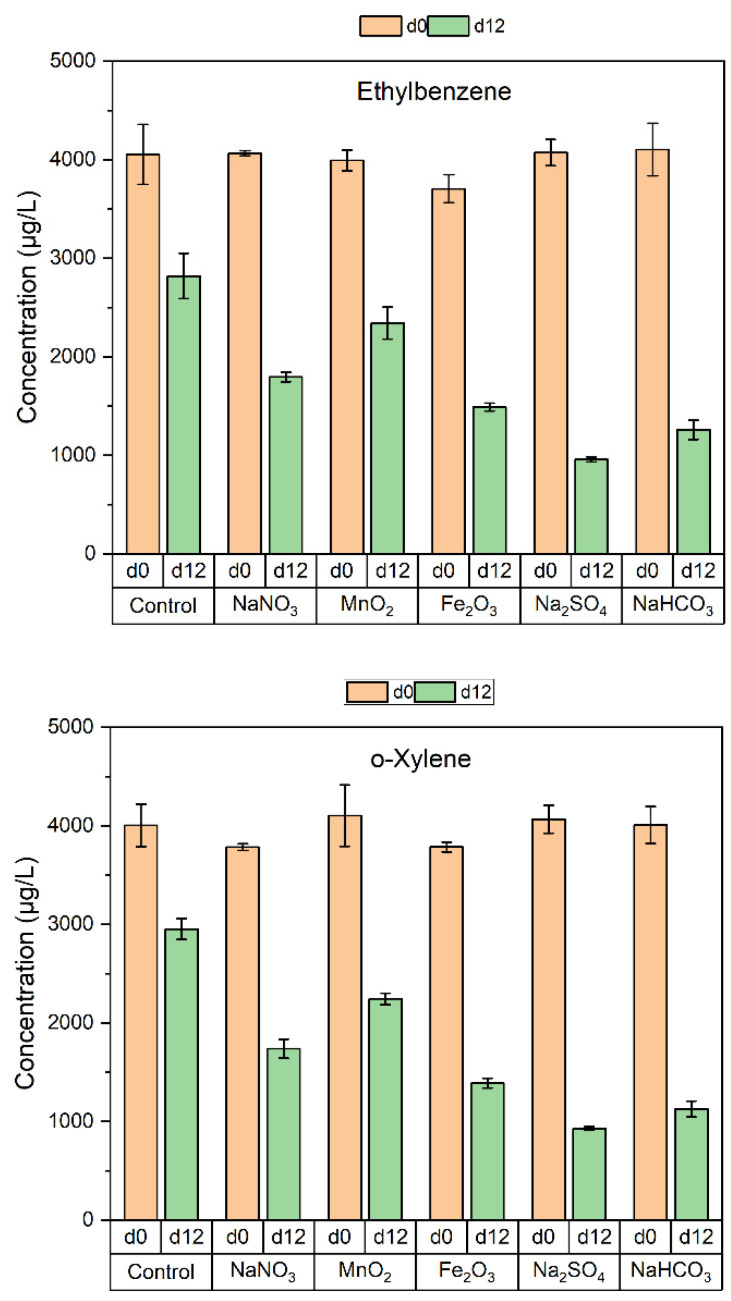
Comparisons of BTEX concentrations at the beginning (d0) and end (d12) of the microcosm study in the presence of various electron acceptors.

Microorganisms from the contaminated soil were inoculated in the microcosms and incubated under anaerobic condition in the presence of various electron acceptors. Figure 5 shows the comparisons of BTEX concentrations at the beginning and end of the microcosm study. The changes of BTEX concentrations over time in different microcosms are shown in Figure S3. In all the microcosms, the concentrations of BTEX decreased over time. Although there were fluctuations in the measured concentrations, by day 12, the concentrations of BTEX in the microcosms with the addition of electron acceptors were all lower than those in the control microcosm, indicating that BTEX biodegradations were stimulated by the added electron acceptors. Both zero- and first-order kinetics were used to fit the data. The obtained R^2^ values were similar, ranging from 0.82 to 0.99 and from 0.81 to 0.99, respectively. Table 4 shows the first-order biodegradation rate constants of BTEX in the presence of various electron acceptors. The biodegradation rate constants were calculated by subtracting the decay rate observed in the control from the decay rates observed in the presence of electron acceptors. The decay of BTEX in the control microcosm might be due to volatilization during the sampling and adsorption to the surfaces of microorganisms or the culture vessels. It is also likely that the sterilized microorganism became active after a few days of incubation and degraded the BTEX. Therefore, the calculated biodegradation rate constants shown in Table 4 may underestimate the biodegradation rate constants in the microcosms added with various electron acceptors.

The biodegradation rate constants of BTEX, shown in Table 4, were within the range reported in the literature, where the degradation rate constants of BTEX by microbes under various anaerobic reducing conditions were mostly 0.01 to 0.14/d [10,19,21]. Although the biodegradation of BTEX was thermodynamically more favorable under denitrifying, manganese-reducing, and iron-reducing conditions, the highest biodegradation rate constants were obtained under sulfate-reducing condition in this study. The reason might be that there were more sulfate-reducing bacteria in the culture than other reducing bacteria. Indeed, many species of Clostridia are sulfate-reducing bacteria [35]. Abu Laban et al. reported that *Pelotomaculum* sp. (a species in the family Peptococcaceae belonging to Clostridiales of Clostridia) coupled the degradation of benzene with sulfate reduction [17]. *Pelotomaculum* sp. was detected in the soil used for inoculation in the microcosm study. Members from Gammaproteobacteria could use nitrate, sulfate, iron, and manganese as electron acceptors. In the study of van der Zaan et al., bacteria from the family Peptococcaceae degraded benzene, and the produced hydrogen was used by bacteria from families Rhodocyclaceae and Burkholderiaceae (both belonging to Betaproteobacteriales of Gammaproteobacteria) for the reduction of nitrate, sulfate, and iron [10]. Ulrich and Edwards reported that *Azoarcus* sp. and *Dechloromonas* sp. (both in the family Rhodocyclaceae) coupled the degradation of benzene with nitrate [10]. Dorer et al. reported that *Aromatoleum aromaticum* EbN1 and *Georgfuchsia toluolica* G5G6 (both in the family Rhodocyclaceae) degraded toluene and ethylbenzene under nitrate-, manganese-, and iron-reducing conditions [19]. In this study, both Rhodocyclaceae and Burkholderiaceae were detected in the soil sample.

In this study, under all the reducing conditions, benzene was degraded slightly faster than the other compounds. Dorer et al. studied the biodegradation of toluene and ethylbenzene by two strains of bacteria under manganese-reducing condition and found that *Aromatoleum aromaticum* EbN1 degraded ethylbenzene faster than toluene, whereas *Georgfuchsia toluolica* G5G6 degraded toluene faster than ethylbenzene [19]. This indicated that the degradation of contaminants varied with bacteria. In the above-mentioned study, toluene and ethylbenzene were studied separately, not like in this study, where BTEX were studied as a mixture. Substrate interactions during anaerobic biodegradation of BTEX by mixed cultures under nitrate-reducing conditions were reported by Dou et al. [36]. In their study, the amendment of toluene or ethylbenzene could stimulate benzene degradation. The simultaneous presence of toluene and ethylbenzene could stimulate the degradation of each other. The addition of toluene stimulated o-xylene degradation, whereas the amendment of ethylbenzene inhibited the degradation of o-xylene. The amendment of benzene would inhibit the degradation of other BTEX compounds. When supplied as BTEX mixture (with initial concentration of each component ranging from 10–150 mg/L), the degradation rate constants had the following order: toluene > ethylbenzene > o-xylene > benzene. In this study, different results were obtained, with the degradation of benzene being the fastest. This might be due to different initial BTEX concentrations and different microorganisms. At the beginning of this study, each component of BTEX was added into the culture at concentration of 5 mg/L. However, the measured initial concentrations varied from less than 4 to near 5 mg/L, probably caused by different levels of volatilization and adsorption to the bacteria or the vessel. More studies are needed to clarify this phenomenon.

## 4. Conclusions

Both the soil and groundwater sampled from a petrochemical site near the Yangtze river were contaminated with BTEX and other petroleum hydrocarbons. Actinobacteria was dominant in the highly contaminated soil, while Proteobacteria was dominant in the slightly contaminated soil and the groundwater. The relative abundances of Firmicutes, Spirochaetes, and Caldiserica were higher in the highly contaminated soil and groundwater samples compared to those in the corresponding slightly contaminated samples. The relative abundances of predicted functions, such as carbohydrate transport and metabolism, nucleotide transport and metabolism, coenzyme transport and metabolism, amino acid transport and metabolism, etc., in the highly contaminated soil and groundwater samples were higher than those in the corresponding slightly contaminated samples. In microcosms inoculated with the soil microorganisms, biodegradations of BTEX occurred, and the first-order biodegradation rate constants of BTEX in the presence of various electron acceptors had the following order: sulfate (0.08–0.10/d) > sodium bicarbonate (0.07–0.09/d) > ferric oxide ((0.04–0.06/d) > nitrate (0.03–0.05/d) > manganese oxide (0.01–0.04/d).

## Figures and Tables

**Table 1:** Physicochemical properties of the soil samples.

	S1	S2
pH	6.4	6.0
Conductivity (μs/cm)	2790	1210
Total nitrogen (mg/kg)	13	7
Total phosphate (mg/kg)	17	9
C6–C9 ^a^ (mg/kg)	11.57	1049.45
C10–C40 ^a^ (mg/kg)	9.73	87.57
VOC ^b^ (mg/kg)	0.083	138.07
Benzene (mg/kg)	0.005	17.47
Toluene (mg/kg)	0.002	1.06
Ethylbenzene (mg/kg)	0.002	22.41
o-Xylene (mg/kg)	<0.001	0.12
m/p-Xylene (μg/kg)	0.002	10.99

^a^ Total petroleum hydrocarbons C6–C9 and C10–C40; ^b^ volatile organic compounds.

**Table 2:** Physicochemical properties of the groundwater samples.

Parameter	GW1	GW2
pH	7.3	7.2
Conductivity (μs/cm)	749	788
ORP (mV)	−75.7	−93.6
DO (mg/L)	0.9	0.5
NO_2_^−^ (mg/L)	0.01	0.01
S^2−^ (mg/L)	0.09	0.46
Fe (mg/L)	<0.1	0.1
Mn (mg/L)	2.1	6.7
COD (mg/L)	2.5	12.2
VOC (μg/L)	6.85	1355.27
Benzene (μg/L)	<1.00	644.03
Toluene (μg/L)	<1.00	16.70
Ethylbenzene (μg/L)	<1.00	207.79
o-Xylene (μg/L)	<1.00	20.43
m/p-Xylene (μg/L)	<1.00	161.03

**Table 3:** Sequencing results and α diversity indices of the soil and groundwater samples.

Matrix	Sample	Depth (m)	No. of Sequences	Richness ^c^	Shannon_e
Soil	S1	4.0 ^a^	33,155	1387	4.41
Soil	S2	4.0 ^a^	49,236	843	2.23
Groundwater	GW1	3.4 ^b^	83,438	1623	4.19
Groundwater	GW2	2.9 ^b^	76,320	1316	3.96

^a^ Depth of soil below the ground; ^b^ depth of water level below the ground; ^c^ equal to number of OTUs.

**Table 4:** Biodegradation rate constants of BTEX in the presence of various electron acceptors (/d).

Compound	Electron Acceptor				
	NaNO_3_	MnO_2_	Fe_2_O_3_	Na_2_SO_4_	NaHCO_3_
Benzene	0.05	0.03	0.06	0.10	0.09
Toluene	0.03	0.04	0.04	0.08	0.08
Ethylbenzene	0.04	0.01	0.05	0.09	0.07
o-Xylene	0.03	0.02	0.05	0.09	0.08

## Data Availability

The data presented in this study are available on request from the corresponding author.

## Supplementary Materials

The following supporting information can be downloaded at: https://www.mdpi.com/article/10.3390/ijerph192416449/s1, Table S1. The components of vitamin solution; Table S2. The components of mineral solution; Figure S1. COG Functional prediction at L2 level for the samples. (S indicates soil, GW indicates groundwater); Figure S2. Pathway of ethylbenzene degradation (KEGG, 2022); Figure S3. The changes of BTEX concentrations over time in different microcosms.
