# Supplementary material for: Evaluation of Biodegradation of BTEX in the Subsurface of a Petrochemical Site near the Yangtze River, China
Source: Int J Environ Res Public Health. 2022 Dec 8;19(24):16449. doi: 10.3390/ijerph192416449 (PMC9778668; doi:10.3390/ijerph192416449)
Supplement: Supplementary file 1 [file ijerph-19-16449-s001.zip › ijerph-2034540-supplementary.pdf]

Supplemental Materials

Table S1. The components of vitamin solution

Table S2. The components of mineral solution

Figure S1. COG Functional prediction at L2 level for the samples. (S indicates soil, GW indicates groundwater)

Figure S2. Pathway of ethylbenzene degradation (KEGG, 2022)

Figure S3. The changes of BTEX concentrations over time in different microcosms

Table S1. The components of vitamin solution

Compounds	Concentration (g/L)
Nitrilotriacetic acid	1.5
$MgSO_4 \cdot 7H_2O$	3.0
$MnSO_4 \cdot H_2O$	0.5
NaCl	1.0
$FeSO_4 \cdot 7H_2O$	0.1
$CuSO_4 \cdot 5H_2O$	0.01
AlK(SO4)$_2$	0.01
H_3BO_3	0.01
$Na_2MoO_4 \cdot 2H_2O$	0.01
$CuSO_4 \cdot 5H_2O$	0.02
$NiCl_2 \cdot 6H_2O$	0.01
pH	7.0

Table S2. The components of mineral solution

Compounds	Concentration (mg/L)
Biotin	2.0
Folic acid	2.0
Pyridoxine hydrochloride	10.0
Thiamine HCl	5.0
Riboflavin	5.0
Nicotinic acid	5.0
Calcium D-(+)-pantothenate	5.0
Vitamin B12	0.1
p-Aminobenzoic acid	5.0
Thioctic acid	5.0

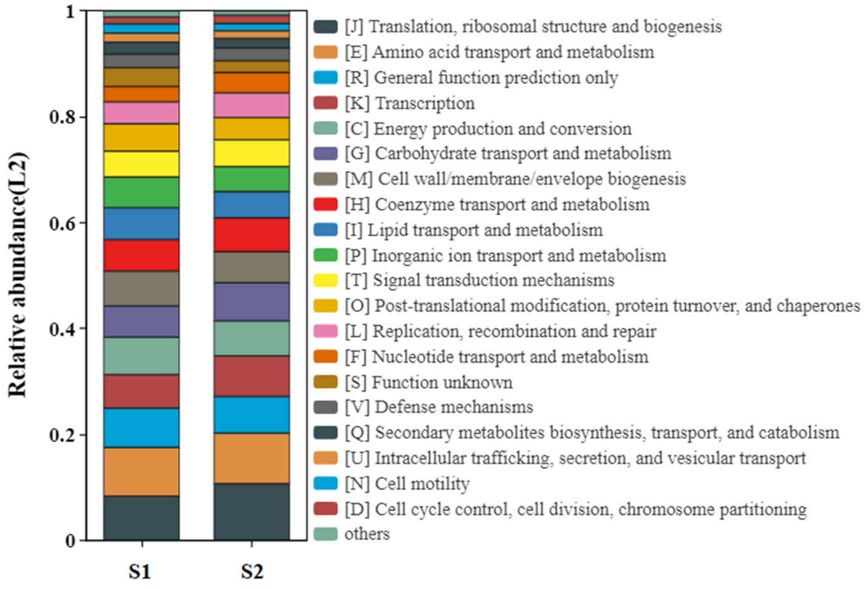

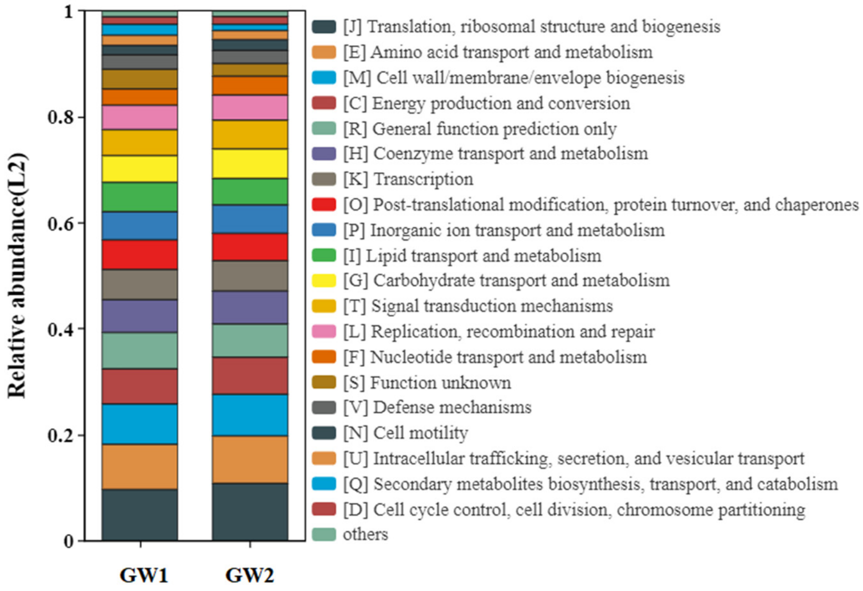

Figure S1. COG Functional prediction at L2 level for the samples.

(S indicates soil, GW indicates groundwater)

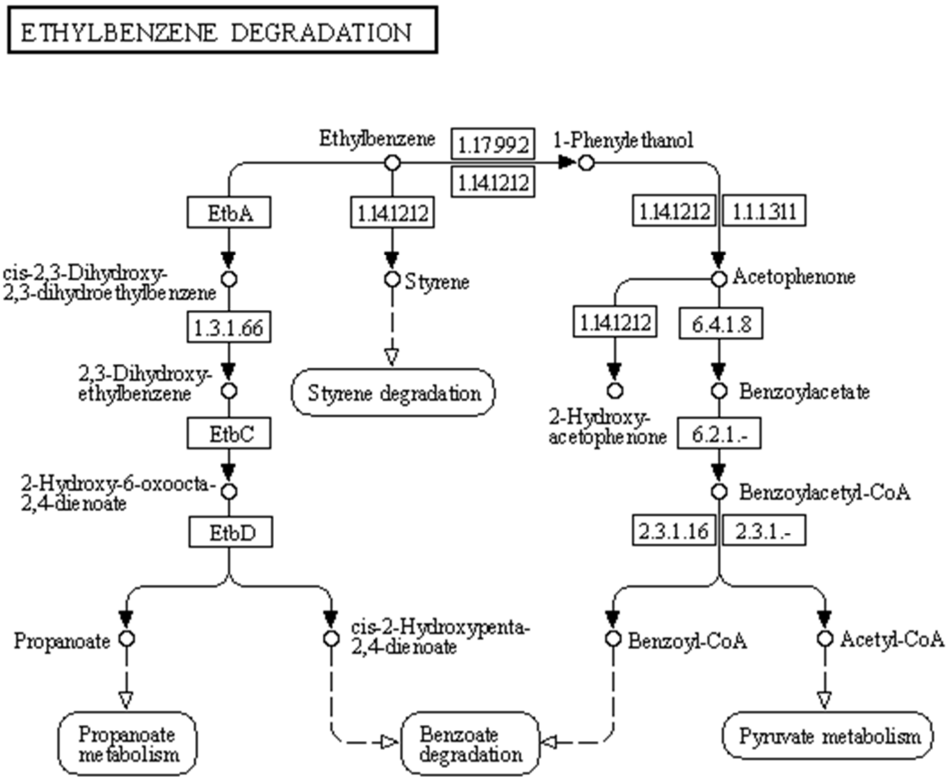

Figure S2. Pathway of ethylbenzene degradation (KEGG, 2022)

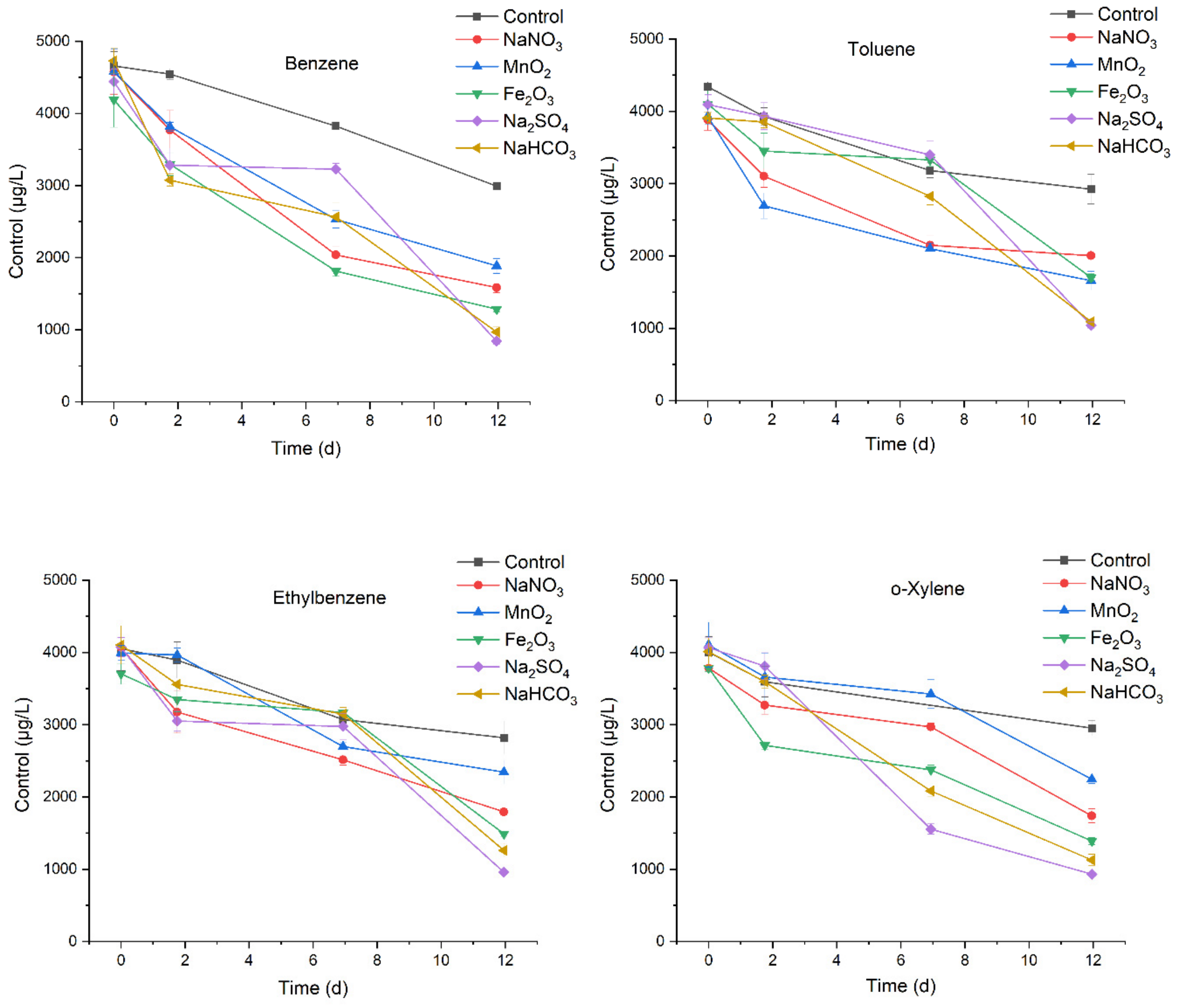

Figure S3. The changes of BTEX concentrations over time in different microcosms
